# Proteomic signatures of 16 major types of human cancer reveal universal and cancer-type-specific proteins for the identification of potential therapeutic targets

**DOI:** 10.1186/s13045-020-01013-x

**Published:** 2020-12-07

**Authors:** Yangying Zhou, T. Mamie Lih, Jianbo Pan, Naseruddin Höti, Mingming Dong, Liwei Cao, Yingwei Hu, Kyung-Cho Cho, Shao-Yung Chen, Rodrigo Vargas Eguez, Edward Gabrielson, Daniel W. Chan, Hui Zhang, Qing Kay Li

**Affiliations:** 1 Department of Pathology, Johns Hopkins Medical Institutions, Baltimore, MD 21231 USA; 2 Department of Chemical and Biomolecular Engineering, Johns Hopkins University, Baltimore, MD 21218 USA; 3 Department of Oncology, Sidney Kimmel Cancer Center at Johns Hopkins Medical Institutions, Baltimore, MD 21224 USA; 4 Department of Urology, Johns Hopkins University, Baltimore, MD 21287 USA

**Keywords:** Proteomic analysis, Data-independent acquisition, Tissue-enriched proteins, Cancer-associated proteins, Cancer therapeutic targets

## Abstract

**Background:**

Proteomic characterization of cancers is essential for a comprehensive understanding of key molecular aberrations. However, proteomic profiling of a large cohort of cancer tissues is often limited by the conventional approaches.

**Methods:**

We present a proteomic landscape of 16 major types of human cancer, based on the analysis of 126 treatment-naïve primary tumor tissues, 94 tumor-matched normal adjacent tissues, and 12 normal tissues, using mass spectrometry-based data-independent acquisition approach.

**Results:**

In our study, a total of 8527 proteins were mapped to brain, head and neck, breast, lung (both small cell and non-small cell lung cancers), esophagus, stomach, pancreas, liver, colon, kidney, bladder, prostate, uterus and ovary cancers, including 2458 tissue-enriched proteins. Our DIA-based proteomic approach has characterized major human cancers and identified universally expressed proteins as well as tissue-type-specific and cancer-type-specific proteins. In addition, 1139 therapeutic targetable proteins and 21 cancer/testis (CT) antigens were observed.

**Conclusions:**

Our discoveries not only advance our understanding of human cancers, but also have implications for the design of future large-scale cancer proteomic studies to assist the development of diagnostic and/or therapeutic targets in multiple cancers.

## Introduction

Great efforts have been made to construct a comprehensive genomic landscape of human cancers using large-scale genomic data [1, 2]. These studies, particularly the Cancer Genome Atlas (TCGA) project, focus on the discovery of the cellular origin and oncogenic processes of cancers [3–6]. These greatly advance our knowledge in cancer biology, cancer screening and diagnosis, and also facilitate the development of targeted- and immuno-therapies [7–9]. However, the expression of protein-coding genes as well as the regulation of intracellular signaling networks is a dynamic and integrative process of genomic, transcriptomic, and translational events [10, 11]. It is unclear to what extent of these molecular alterations are translated into proteins. Previous studies have also shown proteome variations in different tissues [12–14]. Therefore, a comprehensive proteomic-based analysis of major cancer types can provide information beyond genomics and further aid in the discovery of molecular signatures in cancers to understand the functional consequences of certain key genomic aberrations.

Recently, data-independent acquisition (DIA) of mass spectrometry (MS), also called Sequential Window Acquisition of all THeoretical fragment ion spectra (SWATH), has emerged as an alternative technology for proteomic analysis of biological samples to minimize the data-dependent acquisition (DDA)-based analytic limitations, for instance, the stochastic nature of precursor ion selection and low sampling efficiency [15–19]. DIA is an unbiased methodology that allows peptide precursor ions divided into several consecutive windows during fragmentation resulting in a comprehensive fragmentation map of all detectable precursors for accurate quantification of the given sample. A recent study involving 11 institutions worldwide has demonstrated that DIA (SWATH-MS) is a fast, simple and reproducible method for large-scale proteomic quantitative analysis [17]. Other benefits of using DIA-MS based technology include requiring less quantity of clinical samples and providing sufficient proteome coverage with quantitative consistency and analytic accuracy [20, 21].

To facilitate and advance our understanding in human cancers from proteomic perspective, we extensively analyzed 16 major types of treatment-naïve primary human cancer (including cancerous and non-cancerous tissues) using DIA-MS. Our proteomic-based approach has characterized major human cancers and identified universally expressed proteins as well as tissue-type-specific and cancer-type-specific proteins. Additionally, our study provides new insights into potential therapeutic and diagnostic targets.

## Methods

### Tissue sample acquisition

All tissue samples and associated clinical information were obtained with approval from the Institutional Review Board of Johns Hopkins Medical Institution under informed consent. Tissue specimens were collected from cancer patients diagnosed at Johns Hopkins Hospitals, whose tumors were untreated and underwent surgical resection. Each tissue sample had less than 30 min of cold ischemia time after resection and was flash-frozen in liquid nitrogen. All cases were reviewed by the American Board of Pathology certified pathologists to confirm the morphological diagnosis and tumor staging. Patients with prior history of other malignancies in the past 12 months, any systemic chemotherapy, endocrine or immune-related therapy, as well as prior radiation therapy for any cancer type, were excluded from this study.

In total, 246 tissue samples of 16 cancer types from 141 patients were collected for this study with an average age of 64 years (ranged from 27 to 92 years old). Our cohort had a relatively balanced sex distribution with 73 males and 68 females. The pathological stage of cancers was classified according to the American Joint Committee on Cancer (AJCC) staging manual and World Health Organization (WHO) criteria [22]. Patients’ demographics, tumor histopathologic characteristics, and other clinical information are summarized in Additional file 2: Table S1.

### Liquid chromatography tandem mass spectrometry (LC–MS/MS) Analysis

The detailed procedure of protein extraction, trypsin digestion, and peptide desalting of tissue samples is described in Additional file 1: Supplementary Material.

The DIA-MS analysis was performed using a Q-Exactive HF-X mass spectrometer connected to an EASY-nLC 1200 system (Thermo Scientific, USA). All individual tumor, NAT and normal samples were resuspended in 3% ACN / 0.1% FA with indexed retention time (iRT) peptides (Biognosys, Zurich, Switzerland) adding into each sample according to manufacturer’s instructions for DIA MS analysis. About 1 μg of peptides was loaded onto a 28-cm-long self-packed C18 column (1.9 μm/120 Å ReproSil-Pur C18 resin, Dr. Maisch GmbH, Germany) with an integrated PicoFrit emitter (New Objective, Inc., USA). Mobile phase flow rate was 300 nL/min with buffer A (0.1% FA) and buffer B (0.1% FA, 80% ACN). A 120-min gradient was performed as follows: 2% B in 1 min, from 2 to 30% B for 94 min, increase to 60% B for 9 min, ramp to 90% B in 1 min, and held at 90% B for 5 min, then drop to 50% in 1 min, and held at 50% for 9 min. The full MS1 scan was acquired from a range of 400–1000 m/z at a resolution of 120, 000, followed by automatic gain control (AGC) set at 1 × 10^6^ and max injection time of 60 ms. For each DIA MS2 scan, the precursor mass range was 400–1000 m/z with a set of 50 overlapping windows, followed by a 12 m/z fixed isolation width, normalized collision energy (NCE) of 30%, and maximum injection time of 25 ms. The resolution and AGC were the same as the MS1 scan.

### Generation of Multi-Cancer spectral library

A Multi-Cancer spectral library was generated by searching DIA data of 246 individual samples and publicly available DDA data downloaded from Human Proteome Map [23] against the human protein sequences from UniProt/Swiss-Prot (released on March 2018) appended with iRT peptide sequences via Pulsar algorithm embedded in Spectronaut Pulsar X (Biognosys, Zurich, Switzerland). The parameter settings for the database search were used as follows. Mass tolerance of MS1 and MS2 was set as dynamic with the correction factor of one. Fragment ions were selected from a range of 300 to 1800 m/*z*, and fragments per peptide were restricted from 3 to 6. A false discovery rate (FDR) was set as 1% to generate the final spectral library. Finally, a spectral library was configured to contain high-quality MS assays of 13,029 proteins and 245,816 peptides.

### Sample quality control

To evaluate quantification accuracy and data reproducibility, we measured the performance of one sample from our cohort at three different time points (three injections each time point)—in the beginning, in the middle, and at the end of the entire DIA-MS data acquisition for data quality control. The reproducibility was evaluated and computed using the coefficient of variations (CV) and pair-wise Pearson correlation among nine injections.

### DIA data analysis

For the quantitative analysis of proteins across the 246 tissue samples, DIA raw data files were first searched against the Multi-Cancer spectral library followed by the quantification via Spectronaut Pulsar X. Mass tolerance of MS1 and MS2 were set as dynamic with the correction factor of one. Source-specific iRT calibration was enabled with a local (non-linear) RT regression. Interference correction on MS2 level was enabled, and cross run normalization using the sample median was selected. All quantified proteins were filtered by a *Q* value cutoff of 0.01 (corresponded to an FDR of 1%). For downstream data analyses, we used proteins quantified (after sample assessment) in at least 40% of samples in individual cancer type, where missing values were filled in by either 5% of the lowest intensity (> 100) in a sample if the protein quantified in less than half of the samples with the same tissue type, or averaged intensity minus 2 times the standard deviation (SD) of samples with the same tissue type.

### Sample quantitative assessment

Each sample was assessed quantitatively, where samples in one cancer type were evaluated independently from other cancer types. A sample was qualified for the downstream analyses if it met at least three out of the five following criteria: (1) it had at least 50% of proteins quantified; (2) it had a similar protein median relative to other samples; (3) its correlation with other samples (within the same tissue type) was above 0.6; (4) it had similar protein distribution relative to other samples with the same tissue type, and (5) it was grouped with other samples (same tissue type) in the principal component analysis.

### Tissue-enriched proteomic analysis

For the tissue-enriched proteomic analysis, the normal and tumor tissues were regarded as a single entity in each cancer type. Briefly, pair-wise tissue expression comparison was conducted on *t* test with *p* value adjusted via Benjamini–Hochberg and the log2 fold changes were used to measure the expression difference between tissues. A protein was considered as enriched in a particular type of tissue if the expression difference > 4 folds with an adjusted *p* value < 0.05 compared with 70% of other tissue types. Biological processes of tissue-enriched proteins were based on Gene Ontology annotation via WebGestalt [24].

### Differential proteomic analysis

Differential proteome analysis between tumor and normal samples was conducted. *t* test was performed with *p* value adjusted via Benjamini–Hochberg and log2 fold changes were computed to determine differential abundances of proteins between tumor and normal tissues for each cancer type. Proteins with fold change > 2 and adjusted *p* value < 0.05 were considered to be cancer-associated proteins. Protein annotation (e.g., plasma proteins, secreted proteins) was given by Human Proteome Atlas (HPA, version 19.3, https://www.proteinatlas.org/) [25]. Some cancer-associated proteins were further explored in HPA and Gene Expression Profiling Interactive Analysis (GEPIA, 2019 Release, http://gepia.cancer-pku.cn/index.html) [26] for their protein and mRNA expression levels as reported by these databases when feasible. The heat map was constructed using cancer-associated proteins that the expression values were transformed into *Z* score at the protein level. Four major protein clusters were generated using hierarchical clustering. Biological processes of cancer-associated proteins in each protein cluster were based on Gene Ontology annotation via WebGestalt [24].

### Druggable proteome analysis

Information of drugs and their targets was extracted using dbparser (R package version 1.1) from the XML file (version 5.1.5, www.drugbank.ca/) downloaded from DrugBank [27]. Potential drug targets were further mapped to UniProt [28], PhosphoSitePlus [29], Therapeutic Target Database (TTD) [30], Genomics of Drug Sensitivity in Cancer (GDSC) [31], and HPA to acquire additional information on whether the targets had drug response data, or they were receptors, kinases, or known cancer/FDA-approved/potential drug targets. Differentially expressed proteins in tumor tissues relative to normal tissues (fold change > 2 and adjusted *p* value < 0.05) overlapped with drug targets were further assessed. Cancer drug target candidate proteins were mapped to the Kyoto Encyclopedia of Genes and Genomes (KEGG) pathways via WebGestalt [24].

### Cancer/testis antigens analysis

Information of cancer/testis (CT) antigens was downloaded from CTdatabase [32]. The database contains 269 carefully curated CT antigens with literature-derived information. CT antigens observed only in the current study were further analyzed.

## Results

### Experimental design and proteomic profiling of cancerous and non-cancerous tissues

**Fig. 1:**
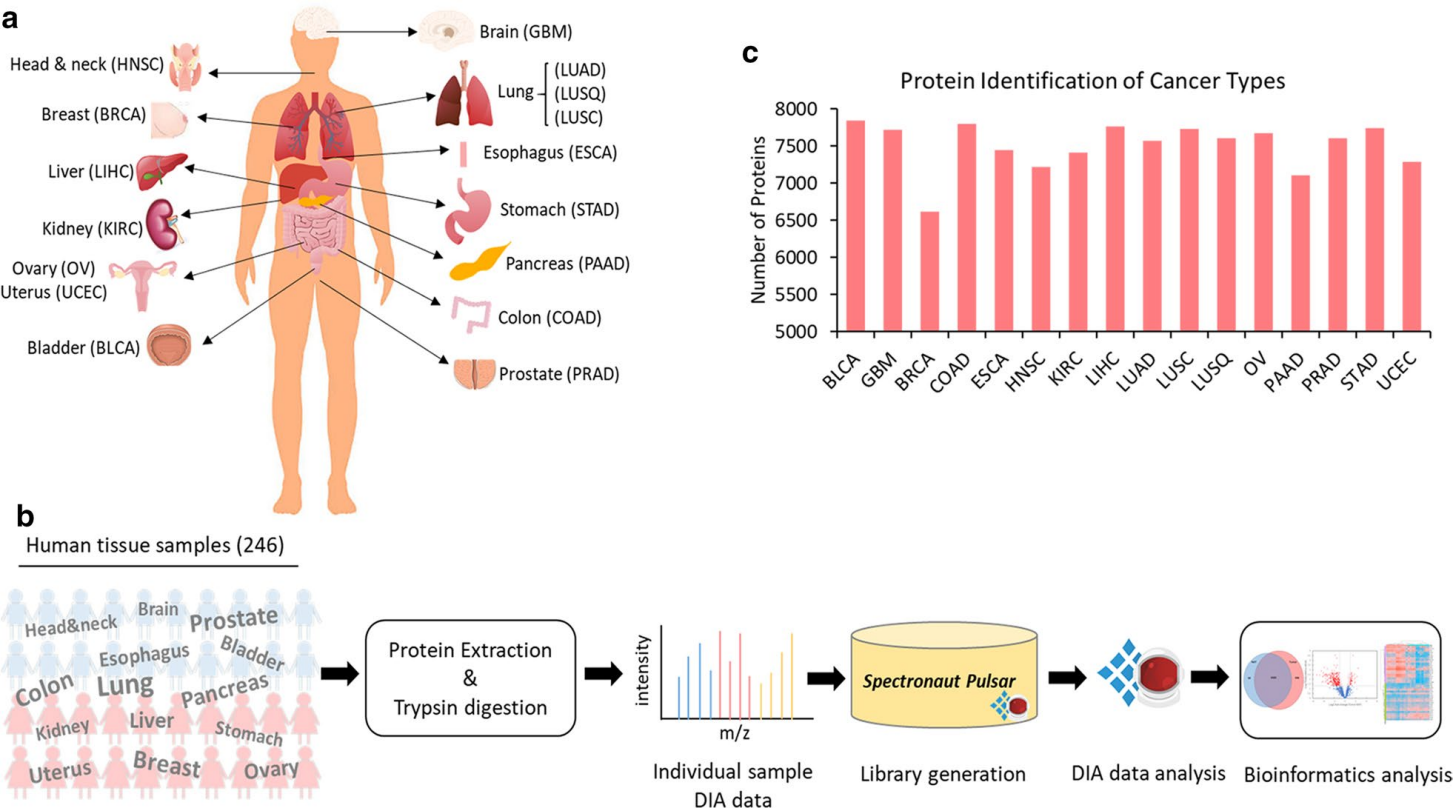
Overview of the clinical cohort and experimental workflow. **a** 16 types of treatment naïve primary human cancers (126 cancer samples, 94 tumor-matched normal adjacent tissues, and 12 normal tissues). Cancer abbreviation annotation: GBM (glioblastoma multiforme), HNSC (head and neck squamous cell carcinoma), LUAD (lung adenocarcinoma), LUSQ (lung squamous cell carcinoma), LUSC (lung small cell carcinoma), ESCA (esophagus squamous cell carcinoma), STAD (stomach adenocarcinoma), PAAD (pancreatic adenocarcinoma), COAD (colon adenocarcinoma), LIHC (liver hepatocellular carcinoma), KIRC (kidney renal clear cell carcinoma), BLCA (bladder urothelial carcinoma), PRAD (prostate adenocarcinoma), BRCA (breast invasive carcinoma), OV (ovarian high-grade serous carcinoma), and UCEC (uterine corpus endometrial carcinoma). **b** Experimental workflow for generating DIA-MS-based proteomic data. **c** The number of proteins identified in each cancer type

The main focus of the current study was to construct proteomic maps of 16 major human cancerous and non-cancerous tissues from a cohort composed of treatment-naïve primary tumor tissues (T), tumor-matched normal adjacent tissues (NAT), and normal tissues (N) (Fig. 1a). The 16 major cancer types along with the number of samples for each and the short tag for each cancer type used in the study are as follows: head and neck squamous cell carcinoma (HNSC, T = 6, NAT = 6), brain glioblastoma, which is also called glioblastoma multiforme (GBM, T = 12), lung adenocarcinoma (LUAD, T = 10, NAT = 10), lung squamous cell carcinoma (LUSQ, T = 8, NAT = 8), lung small cell carcinoma (LUSC, T = 6, NAT = 6), esophageal squamous cell carcinoma (ESCA, T = 7, NAT = 7), stomach adenocarcinoma (STAD, T = 9, NAT = 9), pancreatic adenocarcinoma (PAAD, T = 8, NAT = 8), colon adenocarcinoma (COAD, T = 11, NAT = 11), liver hepatocellular carcinoma (LIHC, T = 8, NAT = 8), kidney clear cell renal cell carcinoma (KIRC, T = 10, NAT = 10), bladder urothelial cell carcinoma (BLCA, T = 11, NAT = 11), prostate adenocarcinoma (PRAD, T = 7, N = 7), breast invasive ductal carcinoma(BRCA, T = 5, NAT = 5), ovarian high-grade serous carcinoma (OV, T = 7, NAT = 2, N = 5), and uterine endometrioid adenocarcinoma (UCEC, T = 4, NAT = 4). Patients’ clinical features, including age, gender, race, tumor grade and pathological stage, as well as smoking status are summarized in Additional file 2: Table S1.

Proteomic profiles of all tissue samples were generated using DIA-MS analytic platform as shown in Fig. 1b. Briefly, proteins were extracted from each specimen followed by trypsin digestion to obtain peptides, which were analyzed using a Q-Exactive HF-X mass spectrometer. To perform the quantitative proteomic analysis, DIA raw files of all the samples were searched against a customized spectral library constructed using DIA data of individual samples combined with DDA data from the Human Proteome Map and quantified via Spectronaut.

To control longitudinal data reproducibility through the data acquisition, we performed the measurement of one sample set at the beginning, in the middle, and toward the end of the DIA-MS acquisition for data quality control (QC) (Additional file 2: Table S1). We observed the median coefficient of variations (CV) of the proteins among triplications to be less than 10% (Additional file 1: Figure S1a). We also obtained Pearson correlation coefficients above 0.90 among the injections (Additional file 1: Figure S1b), indicating high reproducibility of the DIA-MS workflow in the quantification of the DIA proteome data in this study.

Prior to downstream analyses, we evaluated the data quality of each clinical sample. Initially, a total of 246 clinically annotated samples, including 129 tumor tissues, 105 NATs, and 12 normal tissues, were included in our study. Two cases of the metastatic tumor were removed, which were initially considered as primary cancers (1 paired tumor/NAT from LUAD and 1 paired tumor/NAT from STAD). By further quantitatively assessing the quality of the remaining 242 samples, ten samples (1 tumor from KIRC and 9 NATs comprised of 3 samples from PAAD and 1 sample each from ESCA, HNSC, LUAD, LUSQ, OV, and STAD) were excluded due to sample disqualification (see method section for details) (Additional file 2: Table S1).

Overall, proteomic profiles of 232 samples, including 126 tumors, 94 NATs, and 12 normal tissue samples were utilized for the comprehensive proteome characterization of 16 major human cancers. A total of 8527 proteins were identified in which the average of 7505 proteins were identified for each cancer type (Fig. 1c). By combining proteins quantified in at least 40% of the samples from each cancer type, 7947 proteins (i.e., quantifiable proteins) were used for downstream analyses (Additional file 3: Table S2). For simplicity, all the NATs and normal tissues referred to as normal tissues in the analyses.

### Characterization of universally expressed proteins in different cancerous and non-cancerous tissues

It is well known that housekeeping genes are a group of genes required in the maintenance of basic biological functions of cells which are consistently expressed in almost all tissues and cells [33, 34]. Although genome-wide profiling of housekeeping genes has been reported in various studies, expression profiles of such genes at protein-level are not available yet. Furthermore, it is unclear to what extent of these genes are transcribed and then translated into proteins, particularly in cancers. Therefore, we analyzed the expression patterns of quantified proteins in tumor and normal tissues.

**Fig. 2:**
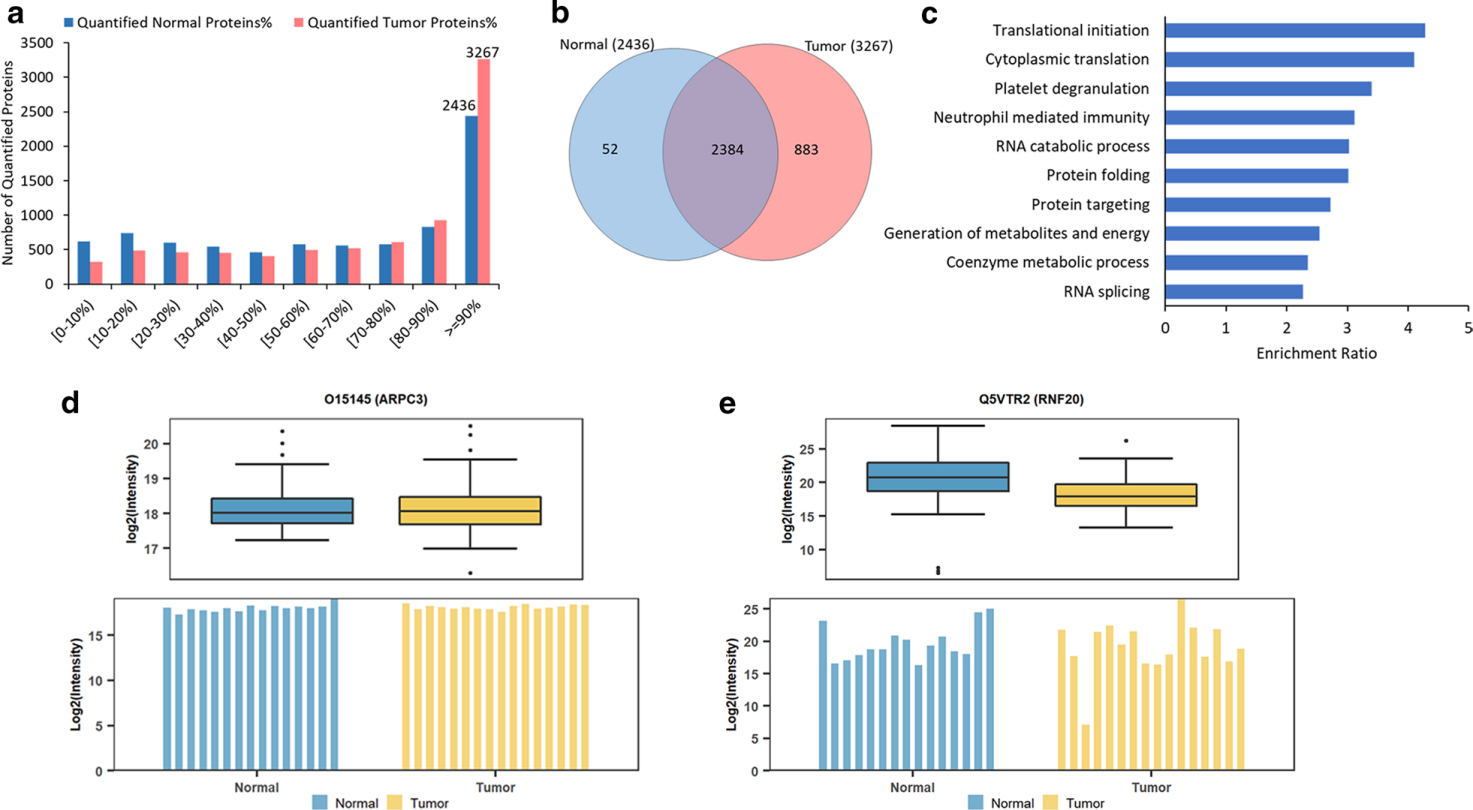
Proteome analysis of housekeeping proteins. **a** The number and percentage of protein quantified in tumor and normal tissues. **b** Proteins quantified in over 90% of normal and tumor tissues, where 2384 commonly observed proteins were considered as housekeeping proteins. **c** Gene ontology biological process analysis of the 2384 housekeeping proteins. **d** A relatively consistent expressional level of ARPC3 across tumor and normal tissues. **e** A fluctuated expressional level of RNF20 across tumor and normal tissues

Our data demonstrated that 3267 and 2436 proteins were quantified in more than 90% of tumor and normal tissues, respectively (Fig. 2a, Additional file 4: Table S3). Among these proteins, 2384 proteins were detected in both normal and tumor tissues (Fig. 2b, Additional file 4: Table S3), which we considered these universally expressed proteins as housekeeping proteins and referred to as universal proteins in this study. Further analysis indicated that the majority of these highly expressed housekeeping proteins belonged to certain intracellular groups, including cytoskeletal proteins, ribosomal proteins, cytosolic proteins, and metabolic enzymes. They were also involved in the translational activity, RNA splicing and catabolism, protein synthesis, cell metabolism, and energy generation (Fig. 2c).

Among the 2384 housekeeping proteins, we found 1934 proteins corresponding to the housekeeping protein-coding genes in the Human Proteome Atlas (HPA, version 19.3) [25], Additional File 1: Figure S2b), such as glyceraldehyde-3-phosphate dehydrogenase (GAPDH), beta-actin (ACTB), and beta-tubulin (TUBB) (Additional file 1: Figure S2a), demonstrated a high consistency between our data and the transcriptomics results. Moreover, the majority of the housekeeping proteins were expressed at relatively similar levels across human cancerous and non-cancerous tissues as expected. For example, the actin-related protein 2/3 complex subunit 3 (ARPC3), a protein participates in the regulation of nuclear actin polymerization and cytoskeleton component [35] (Fig. 2d), and the thioredoxin like 1 (TXNL1), a protein involved in the protein disulfide oxidoreductase activity (Additional file 1: Figure S2c).

However, we also found that several housekeeping proteins with various abundances across cancerous and non-cancerous tissues. For example, the ring finger protein 20 (RNF20) is involved in protein metabolism and ubiquitination; and its expression profiles were inconsistent and relatively low in the majority of tumor tissues compared to normal tissues (Fig. 2e). Nonetheless, our observation was consistent with the previous study, where RNF20 was associated with inflammation activities and down-regulated in multiple cancers [36]. Thus, our findings not only correspond with the transcriptomics results but also suggest a dynamic expression of housekeeping proteins in human cancers.

### Identification of tissue-enriched proteins

The expression of cellular proteins varies in different tissues and organs, as well as in different physiological and pathological conditions [37]. To investigate the tissue-enriched proteins, we performed a pairwise comparison of quantifiable proteins among 16 tissue types. The normal and tumor tissues from each tissue type were considered as a single entity. The protein profile of each tissue type was compared to those generated from others, and proteins with more than fourfold abundance changes (*t* test adjusted *p* value < 0.05) were considered as tissue-type-enriched proteins.

**Fig. 3:**
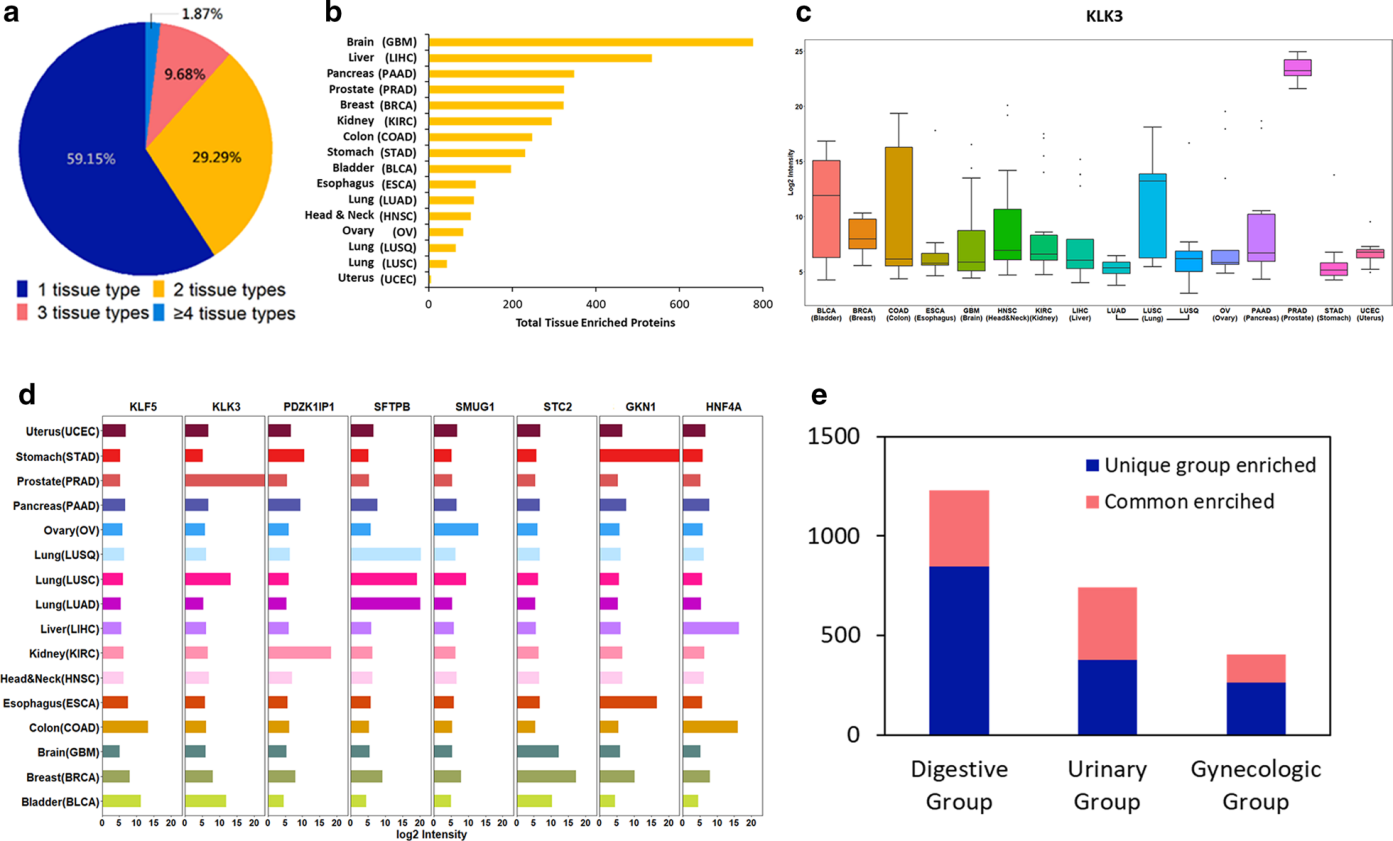
Tissue-enriched proteome analysis. **a** Distribution of tissue-enriched proteins identified in one type of tissue and in multiple types of tissues. **b** The total number of proteins in different tissue types. **c** Expression levels of KLK3 across tumor and normal tissues. **d** Examples of tissue-enriched proteins in different tissue types. **e** Identification of unique group enriched and commonly enriched proteins in digestive, urinary, and gynecologic tissue groups

A total of 2458 tissue-type-enriched proteins were identified (Additional file 5: Table S4). The majority of these proteins were significantly enriched in one tissue type (1454 proteins, 59.15%), followed by two tissue types (720 proteins, 29.29%), three tissue types (238 proteins 9.68%), and four or more tissue types (46 proteins, 1.87%) (Fig. 3a). The number of tissue-type-enriched proteins differed among the tissues. A large number of tissue-type-enriched proteins were found in brain (777 proteins), followed by the liver (535 proteins) and pancreas (348 proteins) (Fig. 3b). Previous studies have shown that proteins enriched in a particular tissue are typically related to specialized functions of that tissue [38]. Indeed, we observed that the majority of tissue-enriched proteins exhibited remarkable differences among the tissues (Additional file 1: Figure S3a). For example, the kallikrein-related peptidase 3 (KLK3), also known as the prostate-specific antigen (PSA), was significantly enriched in the prostate tissue compared to other tissues (Fig. 3c), whereas the surfactant protein B (SFTPB) was significantly enriched in the lung tissue relatively to others (Additional file 1: Figure S3b). We also detected several proteins that were highly enriched in breast, colon, ovary, and kidney, including stanniocalcin-2 (STC2), krueppel-like factor 5 (KLF5), single-strand selective monofunctional uracil DNA glycosylase1 (SMUG1), and PDZK1 interacting protein 1 (PDZK1IP1) (Fig. 3d).

Besides proteins enriched in a particular tissue type, the proteomic patterns of three major tissue groups, namely digestive group (colon, stomach, esophagus, pancreas and liver), urinary group (bladder, prostate and kidney), and gynecologic group (breast, uterus and ovary), also showed unique characterization in certain anatomic sites, where certain proteins were significantly enriched in one tissue group than in the other tissue groups (Fig. 3e, Additional file 5: Table S4 in). For instance, gastrokin-1 protein (GKN1) and hepatocyte nuclear factor 4-alpha (HNF4A) were enriched in digestive group, specifically enriched in the stomach and esophagus (Additional file 1: Figure S3c) and colon and liver (Fig. 3d), respectively.

We further examined the biological processes of tissue-type-enriched and tissue-group-enriched proteins based on Gene Ontology (GO) annotations (Additional file 5: Table S4). The brain was the tissue with the largest number of enriched proteins that the majority of the proteins participated in complex neurological functions, such as neurotransmitter regulation and transport, neuron and axon development, and synaptic signaling transduction. The liver is the tissue with the second largest number of enriched proteins, and most of the proteins were involved in fatty acid metabolism, lipid catabolic process, steroid metabolism, and monosaccharide metabolic process, which were consistent with the metabolic functions of the liver. We also found that proteins enriched in the lung tissue were mainly involved in the regulation of the surfactant homeostasis, cytokine production, and certain immune responses, whereas digestive group-enriched proteins were predominantly involved in enzymatic digestion, glucuronidation, and metabolic and biosynthetic process. Taken together, our results indicate that profiling tissue-enriched proteins can enhance our knowledge of their essential roles in maintaining tissue-specific biological functions.

### Signatures of cancer-associated proteins

We compared the proteomic signatures of the tumor to normal tissues in individual cancer types to characterize cancer-associated proteins. The number of significantly up-regulated and down-regulated proteins (fold change > 2, *t* test adjusted *p* < 0.05) in each cancer type is summarized in Additional file 6: Table S5. Based on our analysis, we found a total of 6835 differentially expressed proteins in at least one cancer type.

**Fig. 4:**
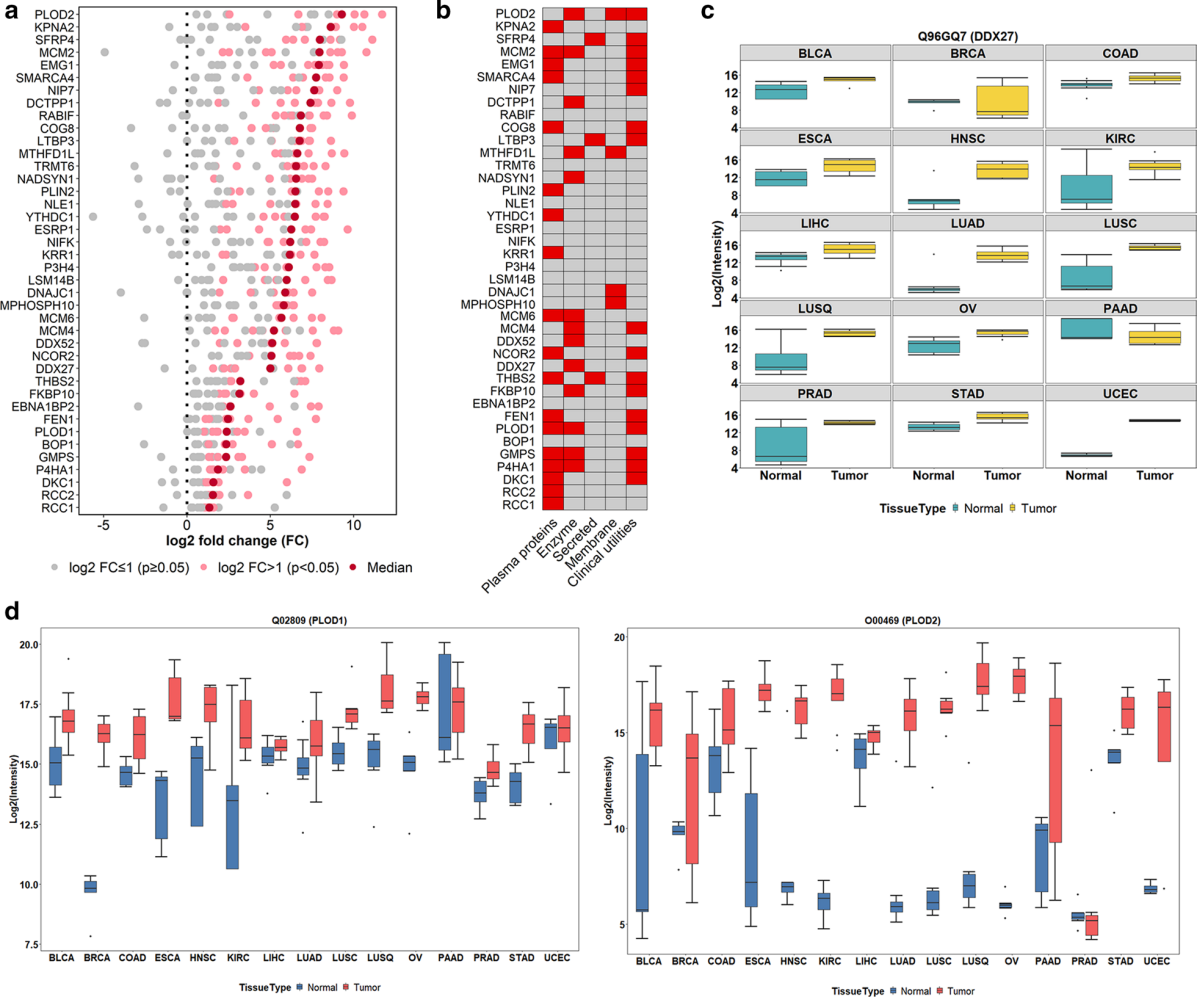
Proteomic analysis of cancer-associated proteins. **a** 40 commonly identified up-regulated proteins between tumor and normal tissues of each cancer type (median in red; log2 FC ≤ 1, adjusted *p* ≥ 0.05 in grey; log2 FC > 1, adjusted *p* < 0.05 in pink). **b** Common cancer-associated proteins annotated by HPA and their known clinical utilities. **c** Expression of DDX27 in tumor and normal tissues across different cancer types (tumor in yellow and normal in green). **d** Expression of PLOD1 and PLOD2 in tumor and normal tissues across different cancer types (tumor in red and normal in blue)

Among the differentially expressed cancer-associated proteins, 40 proteins were significantly elevated in more than 40% of cancer types (Fig. 4a), of which 18 proteins were identified as plasma proteins, three were secretory proteins, four were transmembrane proteins, and 13 were enzymes. According to HPA and DrugBank [27], 17 of these proteins have potential clinical utilities as diagnostic, prognostic markers, or therapeutic targets (Fig. 4b). Up-regulation of DEAD-box helicase 27 (DDX27), a member of DEAD-box RNA helicase superfamily, was found in most cancer types (Fig. 4c). This protein is involved in various biological processes, including ribosome biogenesis, translation, and RNA transport and metabolism [39]. Recent studies have also suggested its multifunctional role in carcinogeneses, such as involvement in the colony-forming of gastric cancer cells, promotion of colorectal cancer growth and metastasis, and the potential usage in the prediction of poor prognosis in gastric and colorectal cancer patients [40, 41]. We also found that the expression of PLOD1 and PLOD2, members of procollagen-lysine, 2-oxoglutarate 5-dioxygenase (PLOD) superfamily, was overexpressed in multiple cancer types (Fig. 4d). Both proteins play a critical role in mediating the formation of stabilized collagen cross-links and promoting tumor progression and metastasis [42]. By calculating the proportion of immuno-staining with high, medium, low, or not detected in different cancer types as reported by HPA, we found DDX27 and PLOD2 showing medium to high tumor-specific staining in multiple cancer types (Additional file 5: Figures S4a-b). We also observed the overexpressed mRNA level of DDX27 and PLOD2 in various cancer types in Gene Expression Profiling Interactive Analysis (GEPIA) (Additional file 5: Figures S4c-d). We found that our results were consistent with HPA and TCGA (from GEPIA) indicating the potential of our data for providing additional information in carcinogenesis. Besides DDX27 and PLOD2, we also found members of minichromosomal maintenance (MCM) superfamily, MCM2, MCM4 and MCM6, were up-regulated across diverse cancer types (Additional file 1: Figure S5) suggesting possible dysregulation in DNA replication and proliferation of cancer cells [43], which might contribute to the development of tumors in these cancer types.

**Fig. 5:**
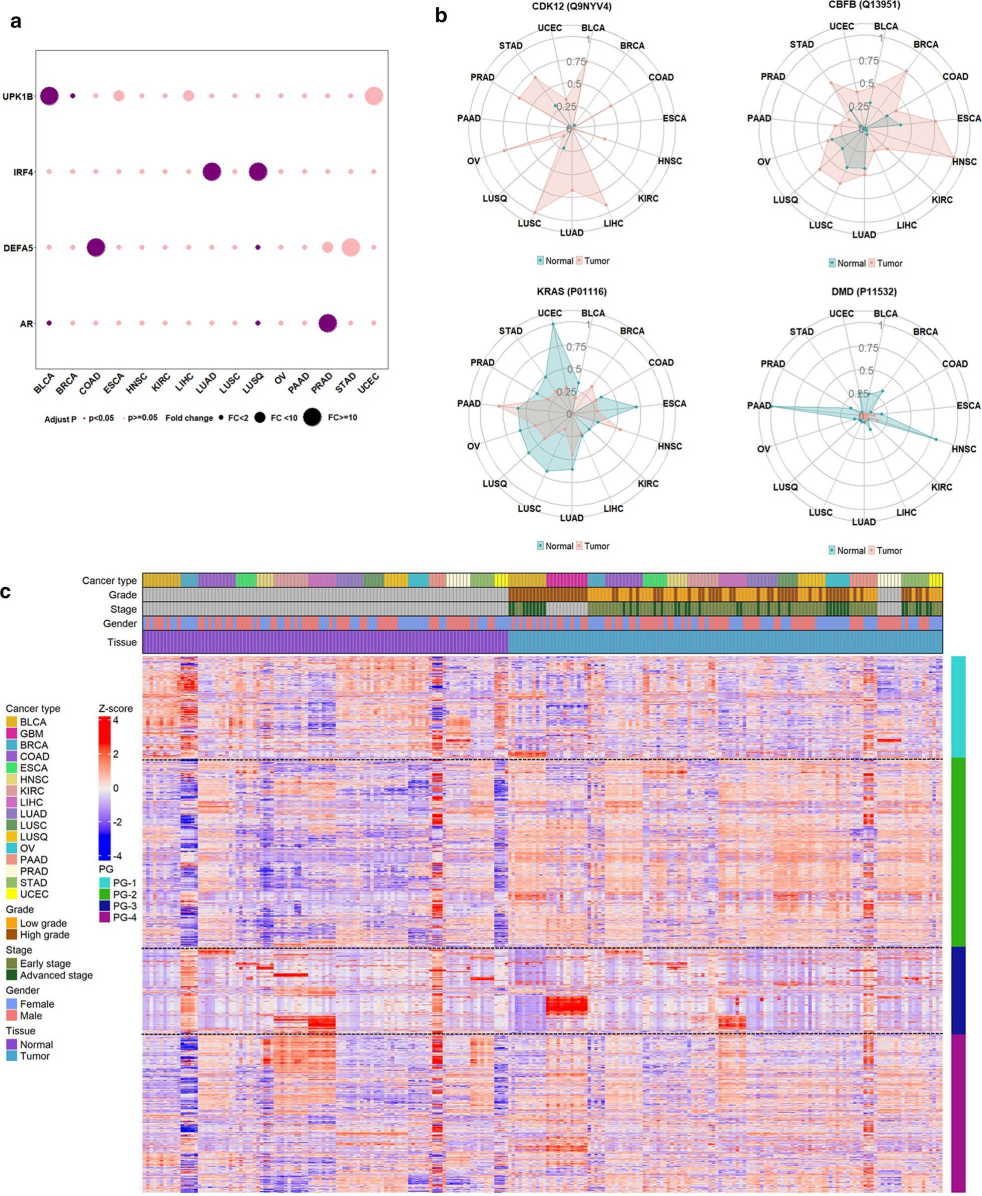
Characterization of cancer-associated proteins. **a** Uniquely expressed proteins in certain cancer types. **b** Expressions of oncoproteins in tumor and normal tissues for different cancer types. **c** A landscape of cancer-associated proteins in 16 cancer types with four major protein groups (PGs) determined via hierarchical clustering

Furthermore, we identified a subset of proteins which were uniquely overexpressed in certain cancer types (Fig. 5a, Additional file 6: Table S5). For example, androgen receptor (AR) significantly elevated in prostate cancer compared with others, consistent with the known role of AR in prostate cancer development and disease progression. Similarly, we found that uroplakin 1B (UPK1B) was overexpressed in bladder cancer, indicating its unique role in bladder tumor development. The defensin alpha 5 (DEFA5), an intestinal tissue-enriched protein, was specifically overexpressed in colon cancer. The interferon regulatory factor 4 (IRF4) was overexpressed in non-small cell lung cancer (NSCLC) including both lung adenocarcinoma and squamous cell carcinoma, but not in lung small cell carcinoma, indicating its potential role for the differentiation of NSCLC from small cell lung cancer.

To examine if any cancer-associated proteins potentially functioned as cancer driver proteins, we mapped the cancer-associated proteins with 299 cancer driver genes selected from the comprehensive analysis of genomic data of cancers [1]. We found that 155 cancer-associated proteins were products of cancer driver genes. Among these proteins, cyclin-dependent kinase 12 (CDK12) and core-binding factor subunit beta (CBFB) were overexpressed in tumors, which were consistent with their oncogenic role in certain types of cancer (Fig. 5b). We also noted that protein levels were not always parallel to the expression of their derived oncogenes in carcinogenesis. For example, oncogene products of KRAS and dystrophin (DMD) proteins demonstrated reduced expressions in several cancer types, in spite of the up-regulated status of these oncogenes (Fig. 5b). Our findings suggested that proteomics data can provide unique information about cancer progression relative to genomic analysis. Other factors such as DNA methylation and transcriptional factors may also play critical roles in protein expression, which need to be further investigated.

Based on the differential protein expression levels between cancerous and non-cancerous tissues, four distinct protein groups (PG) were derived using supervised hierarchical clustering (Fig. 5c, Additional file 6: Table S5). Proteins in Protein Group 1 (PG-1) were highly expressed in most normal tissues including bladder, breast, lung, ovary and uterus. PG-2 proteins were up-regulated in almost all tumor tissues. PG-3 proteins were highly expressed in brain and liver cancer with moderate expression in normal tissues of the breast, liver, kidney, and pancreas. PG-4 proteins were elevated in most cancer types, except for liver and kidney. Moreover, each protein group revealed different biological processes. PG-1 was mainly involved in cell adhesion and coagulation, humoral immune response, and inflammation responses. PG-2 was associated with mRNA processing, RNA splicing and catabolic process, ribonucleoprotein complex organization and localization. PG-3 was involved in the metabolic and biosynthetic processes, such as cellular amino acid, steroid and lipid, sulfur compound, and monosaccharide. PG-4 focused on mitochondrial activity, granulocyte activation, and metabolic processes, such as tricarboxylic acid, ribonucleotide, fatty acid, and coenzyme (Additional file 6: Table S5).

Our data indicated that the reduced or elevated expression levels of cancer-associated proteins may contribute to the dysregulated cellular functions, tumor development, and survival. Overexpression or loss of expression of such proteins may lead to the dysfunction of cancer cells and the overgrowth of tumors. These findings not only broadened the current knowledge of the expression patterns of cancer-associated proteins but also provided new insights into the fields of cancer biomarker discovery.

### Profile of cancer-related druggable proteome

**Fig. 6:**
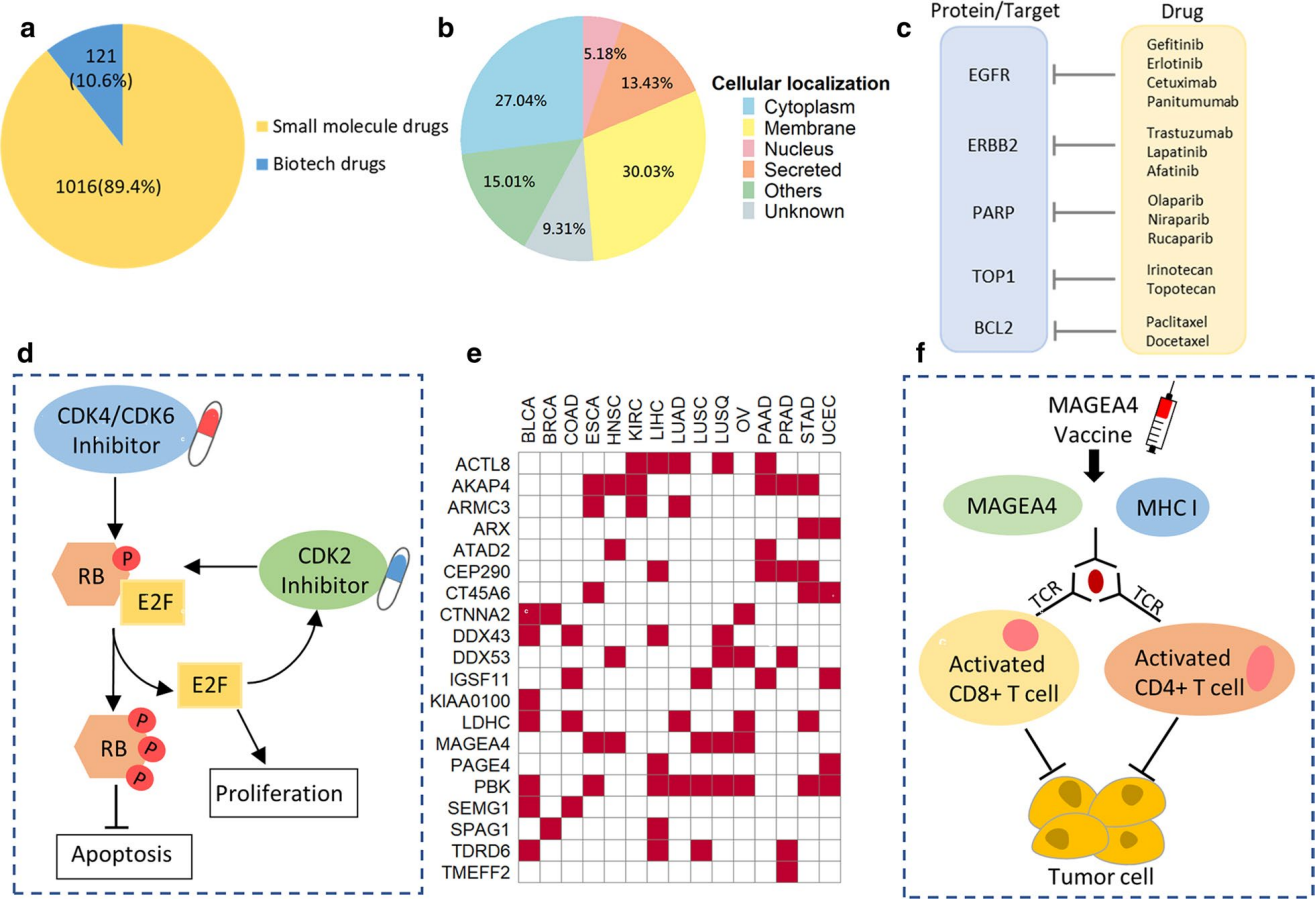
Characterization of cancer-related druggable proteins. **a** Drug types according to the DrugBank. These drugs had targets identified in our proteomic data. **b** Cellular localization of identified druggable proteins. **c** Some identified drug targets with corresponding drugs in this study. **d** A model depicts the CDK2 and CDK4/6 regulating proliferation and apoptosis of tumor cells through retinoblastoma protein (RB) phosphorylation. **e** Elevated expression of cancer/testis (CT) antigens in tumor tissues in at least one cancer type compared to normal tissues. **f** A model shows the MAGEA4 vaccine for the inhibition of tumor cell proliferation

Targeted cancer therapeutics utilize drugs that interfere with the functions of specific genes or proteins in dysregulated pathways to block the growth and spread of cancer [44]. The current era of targeted therapy and immunotherapy has led to the development of precision medicine for cancer patients [45]. Currently, the US Food and Drug Administration (FDA) has approved 2358 anticancer drugs and 4501 anticancer drugs are in phase I*/*II*/*III investigation according to DrugBank [27]. We identified 1139 protein targets corresponding to 1137 therapeutic drugs (including 912 FDA-approved drugs and 91 clinical investigational drugs, Additional file 7: Table S6). Among 1137 therapeutic drugs, 1016 and 121 were categorized as small molecule drugs and biotech drugs in DrugBank, respectively (Fig. 6a). The 1139 drug target proteins were classified into membrane proteins (30.03%), cytoplasmic proteins (27.04%), secreted proteins (13.43%), and nuclear proteins (5.18%, Fig. 6b). By further explored the 1139 drug targets in HPA, Genomics of Drug Sensitivity in Cancer (GDSC) [31], and Therapeutic Target Database (TTD) [30], we found that 67 were cancer drug targets in TTD, including the well-known Epidermal growth factor receptor (EGFR), Receptor tyrosine-protein kinase erbB-2 (ERBB2), and Serine/threonine-protein kinase mTOR (MTOR) (Additional file 7: Table S6). We also mapped to 63 cancer-related FDA-approved drug targets in HPA and 18 proteins with drug response data in GDSC (Additional file 7: Table S6). In addition, we found 85 and 72 potential drug targets were receptors as reported by UniProt [28] and/or kinases as reported by PhosphoSitePlus [29], respectively (Additional file 7: Table S6).

To profile drug targetable candidate proteins, we used the following criteria for filtering: (1) proteins were drug targets of FDA-approved drugs and (2) the proteins were overexpressed in at least one cancer type with more than twofold increase in tumor tissues relatively to normal tissues (*t* test adjusted *p* < 0.05). We identified 464 potential cancer drug target proteins (Additional file 7: Table S6) with FDA-approved drugs. These protein candidates could be further classified into four functional categories for cancer therapy as follows: (1) proteins involved in well-recognized cancer-related pathways, such as PI3K-Akt, ErRB, and NF-κB signaling pathways; (2) proteins related to cellular metabolism and oxidative stress reactions, particularly the dysfunction in metabolic pathways for valine, alanine, glutamine and choline, and oxidative stress of HIF-1 signaling pathway; (3) proteins that participated in cancer development via microorganism infectious pathways, such as hepatitis B, human T-cell leukemia virus 1, and helicobacter pylori infections; and (4) proteins associated with cell adhesion and coagulation including complement and coagulation cascades.

Among the potential druggable targets, we identified proteins such as EGFR, ERBB2, and poly (ADP-ribose) nuclear enzyme polymerase 1 (PARP1) (Fig. 6c, Additional file 7: Table S6). Both EGFR (also known as HER1/ERBB1) and ERBB2 belong to the ERBB family proteins. The overexpression of EGFR or ERBB2 can promote the proliferation, migration, invasion, and angiogenesis of tumor cells, while suppress their apoptosis [46, 47]. By utilizing the tyrosine kinase inhibitors (e.g., gefitinib and erlotinib) or monoclonal antibodies (e.g., cetuximab and trastuzumab), they can inhibit the activities of EGFR/ERBB2 to effectively suppress the tumor cell growth [48, 49]. PARP1 is a nuclear enzyme, and plays a significant role in the maintenance of genome integrity, DNA repair and cell death [50, 51]. The clinical utility of PARP inhibitors, such as Olaparib, Niraparib, and Rucaparib, have made great progress in targeting several cancer types, including ovarian, breast, and prostate cancer [52, 53]. Besides the proteins for targeted therapy, we also found certain chemotherapy drug targets. Irinotecan and topotecan are the anticancer chemotherapy drugs targeting the DNA topoisomerase 1 (TOP1), whereas paclitaxel and docetaxel target the apoptosis regulator Bcl-2 (BCL2) (Fig. 6c). Moreover, we discovered the drugs targeting cyclin-dependent kinases (CDKs), such as CKD2, CKD4 and CKD6, which were overexpressed in several cancer types. It is reported that the CDK4/6 relate to the regulation of the cell-cycle transition through retinoblastoma protein phosphorylation (pRb), while CDK2 contributes to the hyperphosphorylation of pRb and initiates DNA replication subsequently [54, 55] (Fig. 6d). Furthermore, a previous study showed the role of Rb phosphorylation in proliferation and apoptosis of tumor cells revealing the possibility of targeting Rb phosphorylation via CDK2 inhibitors in colon cancer [13], whereas some CDK4/6 inhibitors have been approved for the treatment of breast cancer [56]. Additionally, we also found some proteins potentially having anti-tumor properties, such as annexin A2 (ANXA2) and vascular cell adhesion molecule 1 (VCAM1), but have not been used in cancer drug development. The elevated expression of ANXA2 can contribute to the tumor progression in estrogen receptor (ER) negative breast cancer cell lines [57]; pancreatic tumor progression can be inhibited by blocking VCAM1 [58]. Our findings indicate that our data have the potential to aid the discovery and identify therapeutic drug targets for the treatment of cancers, which need further studies.

We further characterized candidate tumor antigens which were derived from non-mutated cancer/testis (CT) antigens. The DIA global proteomics data identified a total of 21 such CT antigens, whereas 20 of them were elevated in at least one cancer type with more than twofold increase in protein expression compared to normal tissues (Fig. 6d). One of the detected CT antigens, the sperm-associated antigen 1 (SPAG1) was up-regulated in breast and liver cancers, which was consistent with the HPA data (Additional file 1: Figures S6a-b). We also identified that MAGE family member A4 (MAGEA4) was highly expressed in lung and head and neck cancer (Additional file 1: S6c-d). Previous studies have proved that SPAG1 and MAGEA4 participate in the pathogenesis and progression of certain cancers [59, 60], indicating their potential role as drug targets. In addition, SPAG1 and MAGEA4 are immunogenic proteins, where peptides derived from MAGEA4 significantly induced tumor-specific cytotoxic T cell response in vitro and in vivo in human esophageal cancer [61] and SPAG1-induced humoral immune responses in certain cancers [62]. It is of note that for cancer patients whose tumor expressing MAGEA4 and MHC I, MAGEA4 vaccines can induce MAGEA4-specific immune responses to activate the CD4^+^ and CD8^+^ T cell resulting in the inhibition of tumor cell proliferation [63, 64] (Fig. 6f). The usage of MAGEA4 vaccines have been investigated in several clinical trials and provide new insights for vaccine development in cancer immunotherapy.

Our study is able to characterize not only the protein signatures of drug targets, but also signatures of cancer-related CT antigens for potential immunotherapy. These findings provide a new direction of applying proteomics in identifying potential tumor antigens for vaccine development in cancer immunotherapy.

## Discussion

In this study, cancerous and non-cancerous tissues of 16 major cancer types were acquired by DIA-MS to create a proteomic landscape of human cancers. To the best of our knowledge, this is the first study utilizing the DIA-MS technique for multiple human cancer tissues analyses. Our study has demonstrated that the DIA-MS technique could be effectively applied for characterizing global proteomics in large-scale clinical cohorts. Our study characterized the protein expression pattern across 16 different types of cancers as well as profiled the consistently expressed proteins, tissue-enriched proteins and anticancer targetable signatures, which offers the possibilities to investigate cancer-related cellular functions and discovery of cancer-specific proteins for potential diagnostic or therapeutic targets.

We identified 2384 housekeeping proteins, of which a low variability in protein expression across cancerous and non-cancerous tissues was observed in proteins participating in the basic biological and cellular activities, such as GAPDH, ACTB and TUBB. However, there were proteins with inconsistent expression patterns across different tissue types (e.g., RNF20). The fluctuation in the expression patterns of certain housekeeping genes across different tumor types is possible due to the alterations in microenvironment [65] as well as under certain pathological conditions and diseases [66], especially in cancers [67, 68]. Thus, our findings are consistent with the previous studies and allow further investigation of the biological roles of the housekeeping proteins in different tissues and cancers.

Previous study has shown that the differential expression profiles of tissue-enriched proteins are related to the maintenance of essential biological functions [38]. We found 2458 tissue-enriched proteins among diverse tissues. Many of these proteins played critical roles in various cellular functions. For example, proteins enriched in the brain were mainly involved in complex neurological functions, whereas the proteins enriched in the liver predominantly participated in metabolic functions.

In addition of housekeeping and tissue-enriched proteins, we also identified 6835 cancer-associated proteins that could be classified into four different protein groups, demonstrating distinct biological functions in different cancer types. Several overexpressed proteins were identified in multiple cancers, indicating that they could be used for the development of clinical tests to distinguish cancer patients from healthy individuals. On the other hand, proteins uniquely overexpressed in particular cancer could be used as diagnostic markers to differentiate one cancer type from the rest. Furthermore, we observed a discrepancy between oncogenic alteration and protein abundance change among different cancer types, which indicated the necessity of integrative measurement of genomics and proteomics in precision medicine.

Importantly, we identified 464 potential drug targetable proteins including the targets for the FDA-approved drugs that were up-regulated in tumors relative to normal tissues, which could be divided into four major molecular functional categories in cancer therapy. Among the potential drug targets, we identified some well-known targets, including EGFR, ERBB2, and PARP1 as well as CDK2 and CDK4/6, which are related to the regulation of tumor cells development. Notably, we found several CT antigens that were recurrently overexpressed in tumors. These CT antigens are potential cancer-associated biomarkers, which also play an immunogenic role in tumor-specific cytotoxic T cell response and humoral immune responses as demonstrated by MAGEA4 and SPAG1 [61, 62]. Thus, they could serve as potential targets for vaccine development in cancer immunotherapy.

## Conclusions

In summary, our study demonstrated that the DIA-MS technique could be effectively applied for characterizing global proteomics in large-scale clinical cohorts. We profiled a proteome-wide map of 16 major human cancers by identifying the housekeeping proteins, tissue-enriched proteins, cancer-associated proteins, and potentially druggable proteins, which were supplements for various genomic alterations. Our study provides an invaluable resource that may eventually enable further understanding in the cancer development, exploring potential cancer biomarkers, and discovering new therapeutic approaches. Although our results are observational and limited by the sample size, we still find commonality and heterogeneity among different cancers, and they provide novel insights into human cancer proteomics and should be further investigated in cancer research.

## Supplementary Information

**Additional file 1.** Supplementary methods, detailed information of protein extraction, trypsin digestion of tissue samples, and peptide desalting. Figure S1, sample quality control and reproducibility of the DIA data. Figure S2, characterization of housekeeping proteins. Figure S3, proteome analysis of tissue-enriched proteins. Figure S4, protein and mRNA expression from Human Protein Atlas (HPA) and Gene Expression Profiling Interactive Analysis (GEPIA). Figure S5, protein expression of MCM2, MCM4 and MCM6 in different cancer types tumor and normal tissues. Figure S6, protein expression of cancer/testis (CT) antigens according to the Human Protein Atlas (HPA).

**Additional file 2: Table S1**. Patients’ clinical characteristics and replications for data quality control related to Fig. 1 and Figure S1.

**Additional file 3: Table S2**. Protein expression profiling used for all the downstream analyses.

**Additional file 4: Table S3.** Housekeeping proteins identified from tumor and normal samples related to Fig. 2 and Figure S2.

**Additional file 5: Table S4.** Tissue-enriched proteins related to Fig. 3 and Figure S3.

**Additional file 6: Table S5.** Cancer-associated proteins related to Fig. 4, Fig. 5, Figure S4, and Figure S5.

**Additional file 7: Table S6.** Cancer drug targets related to Fig. 6 and Figure S6.

## Data Availability

The datasets used and/or analyzed during the current study are available from the corresponding author on reasonable request.

## Abbreviations

MS: Mass spectrometry
DIA: Data-independent acquisition
CT: Cancer/testis
TCGA: The Cancer Genome Atlas
SWATH: Theoretical fragment ion spectra
DDA: Data-dependent acquisition
AJCC: American Joint Committee on Cancer
WHO: World Health Organization
ACN: Acetonitrile
MeOH: Methanol
TFA: Trifluoroacetic acid
iRT: Indexed retention time
AGC: Automatic gain control
NCE: Normalized collision energy
FDR: False discovery rate
CV: Coefficient of variations
SD: Standard deviation
HPA: Human Proteome Atlas
GEPIA: Gene Expression Profiling Interactive Analysis
TTD: Therapeutic Target Database
GDSC: Genomics of Drug Sensitivity in Cancer
KEGG: Kyoto Encyclopedia of Genes and Genomes
NAT: Normal adjacent tissues
GBM: Glioblastoma multiforme
HNSC: Head and neck squamous cell carcinoma
LUAD: Lung adenocarcinoma
LUSQ: Lung squamous cell carcinoma
LUSC: Lung small cell carcinoma
ESCA: Esophagus squamous cell carcinoma
STAD: Stomach adenocarcinoma
PAAD: Pancreatic adenocarcinoma
COAD: Colon adenocarcinoma
LIHC: Liver hepatocellular carcinoma
KIRC: Kidney renal clear cell carcinoma
BLCA: Bladder urothelial carcinoma
PRAD: Prostate adenocarcinoma
BRCA: Breast invasive carcinoma
OV: Ovarian high-grade serous carcinoma
UCEC: Uterine corpus endometrial carcinoma
QC: Quality control
GO: Gene Ontology
FDA: Food and Drug Administration
EGFR: Epidermal growth factor receptor
ERBB2: Receptor tyrosine-protein kinase erbB-2
MTOR: Serine/threonine-protein kinase mTOR
PARP1: Poly (ADP-ribose) nuclear enzyme polymerase 1
TOP1: Topoisomerase 1
CDK: Cyclin-dependent kinases
pRB: Retinoblastoma protein phosphorylation
ANXA2: Annexin A2
ER: Estrogen receptor
VCAM1: Vascular cell adhesion molecule 1
