# Supplementary material for: Proteomic signatures of 16 major types of human cancer reveal universal and cancer-type-specific proteins for the identification of potential therapeutic targets
Source: J Hematol Oncol. 2020 Dec 7;13:170. doi: 10.1186/s13045-020-01013-x (PMC7720039; doi:10.1186/s13045-020-01013-x)
Supplement: Supplementary file 1 — Additional file 1. Supplementary methods, detailed information of protein extraction, trypsin digestion of tissue samples, and peptide desalting. Figure S1, sample quality control and reproducibility of the DIA data. Figure S2, characterization of housekeeping proteins. Figure S3, proteome analysis of tissue-enriched proteins. Figure S4, protein and mRNA expression from Human Protein Atlas (HPA) and Gene Expression Profiling Interactive Analysis (GEPIA). Figure S5, protein expression of MCM2, MCM4 and MCM6 in different cancer types tumor and normal tissues. Figure S6, protein expression of cancer/testis (CT) antigens according to the Human Protein Atlas (HPA). [file 13045_2020_1013_MOESM1_ESM.docx]

Additional file 1: Supplementary Information for

**Proteomic Signatures of 16 Major Types of Human Cancer Reveal Universal and Cancer-type-specific Proteins for the Identification of Potential Therapeutic Targets**

**Running Title**: Proteomic analysis of 16 types of human cancers

Yangying Zhou^1, †^, T. Mamie Lih^1,†^, Jianbo Pan^1^, Naseruddin Höti^1^, Mingming Dong^1^, Liwei Cao^1^, Yingwei Hu^1^, Kyung-Cho Cho^1^, Shao-Yung Chen^1,2^, Rodrigo Vargas Eguez^1^, Edward Gabrielson^1,3^, Daniel W. Chan^1^, Hui Zhang^1,2,3*^, Qing Kay Li^1,3*^

† These authors contributed equally to this work.

* Corresponding authors. Email: [huizhang@jhu.edu](mailto:huizhang@jhu.edu) (H.Z); [qli23@jhmi.edu](mailto:qli23@jhmi.edu) (Q.K.L).

**This PDF file includes:**

**Supplementary methods**

Protein Extraction and Trypsin Digestion of Tissue Samples

Peptide Desalting

**Figures S1 to S6**

Figure S1. Sample quality control and reproducibility of the DIA data.

Figure S2. Characterization of housekeeping proteins.

Figure S3. Proteome analysis of tissue enriched proteins.

Figure S4. Protein and mRNA expression from Human Protein Atlas (HPA) and Gene Expression Profiling Interactive Analysis (GEPIA).

Figure S5. Protein expression of MCM2, MCM4 and MCM6 in different cancer types tumor and normal tissues.

Figure S6. Protein expression of cancer/testis (CT) antigens according to the Human Protein Atlas (HPA).

**Other additional files for this manuscript include the following:**

**Additional file 2 to 7 (.xlsx)**

Additional file 2: Table S1. Patients’ Clinical Characteristics and Replications for data quality control.

Additional file 3: Table S2. Protein Expression Profiling.

Additional file 4: Table S3. Housekeeping Proteins identified from tumor and normal samples.

Additional file 5: Table S4. Tissue enriched proteins.

Additional file 6: Table S5. Cancer-associated proteins.

Additional file 7: Table S6. Cancer drug targets.

**Supplementary Methods**

**Protein Extraction and Trypsin Digestion of Tissue Samples**

All tissue samples were lysed using 8 M urea lysis buffer (containing 8 M urea, 50 mM Tris (pH 8.0), 75 mM NaCl, 1 mM EDTA, 2 µg/ml aprotinin, 10 µg/ml leupeptin, phosphatase Inhibitor Cocktail 2/3 at 1:100 (v/v) dilutions, 1 mM PMSF, as well as 10 mM NaFand 20 µM PUGNAc) (1). Protein concentration was detected by the bicinchoninic acid (BCA) assay kit (Pierce Biotechnology, Rockford, USA). Then, proteins were reduced by 5mM dithiothreitol (DTT, Pierce Biotechnology, USA) at 37 °C for 1 h and alkylated by 10 mM iodoacetamide (IAA; Sigma-Aldrich, USA) at room temperature for 45 minutes in the dark. Samples were diluted 4-fold with 50mM Tris HCl to decrease the concentration of urea. Adding Lysyl endopeptidase (LysC; Wako Chemicals, USA) in an enzyme/substrate ratio of 1:50 w/w for 2 h at 25 °C. Then trypsin (0.5ug/ul, Promega, Madison, USA) was added at enzyme/protein ratio 1:50 w/w to the solution and incubated at 25°C overnight in the incubator shaker.

**Peptide Desalting**

The C18 column (Sep-Pak tC18 cartridge, Waters, USA) were conditioned three times with 100% Acetonitrile (ACN) and 50% (v/v) ACN / 0.1% (v/v) FA, then equilibrated four times with 0.1% (v/v) trifluoroacetic acid (TFA). The solutions after trypsin digestion were acidified by formic acid (FA) with pH<3. Samples were centrifuged at 13, 000g for 10 min and the supernatant was running through C18 cartridges and desalted three times by 0.1% (v/v) TFA. Finally, the peptides were eluted with 50% (v/v) ACN / 0.1% (v/v) FA. Then we further aliquot 30 μg eluted peptide for further C18 stage-tips desalting.

The C18 stage-tips were prepared using C18 extraction disks (solid-phase C18 extraction disks, diam. = 47 mm, 20 pack; Empore, USA) (2). Three plugs of C18 material were packed into the tips of each stage-tip (200-µl pipette tip) for a total binding capacity of ~30 µg. Using a 16-gauge blunt-end metal needle to hole-punch the ~1mm disks and stabilize the tips (1). Then the packed stage-tips were conditioned twice with 100 μl methanol (MeOH) and 50% (v/v) ACN / 0.1% (v/v) FA, and equilibrated the stage-tips twice with 100 µl of 1% (v/v) FA. After samples loading, stage-tips were washed two times with 100 μl 1% (v/v) FA, and peptides subsequently eluted with 100 μl 50% (v/v) ACN in 0.1% (v/v) FA. Finally, all eluates were dried in a Speed-Vac (Thermo Scientific, USA) and stored at 80 °C until LC-MS/MS analysis.

**Figure S1.**


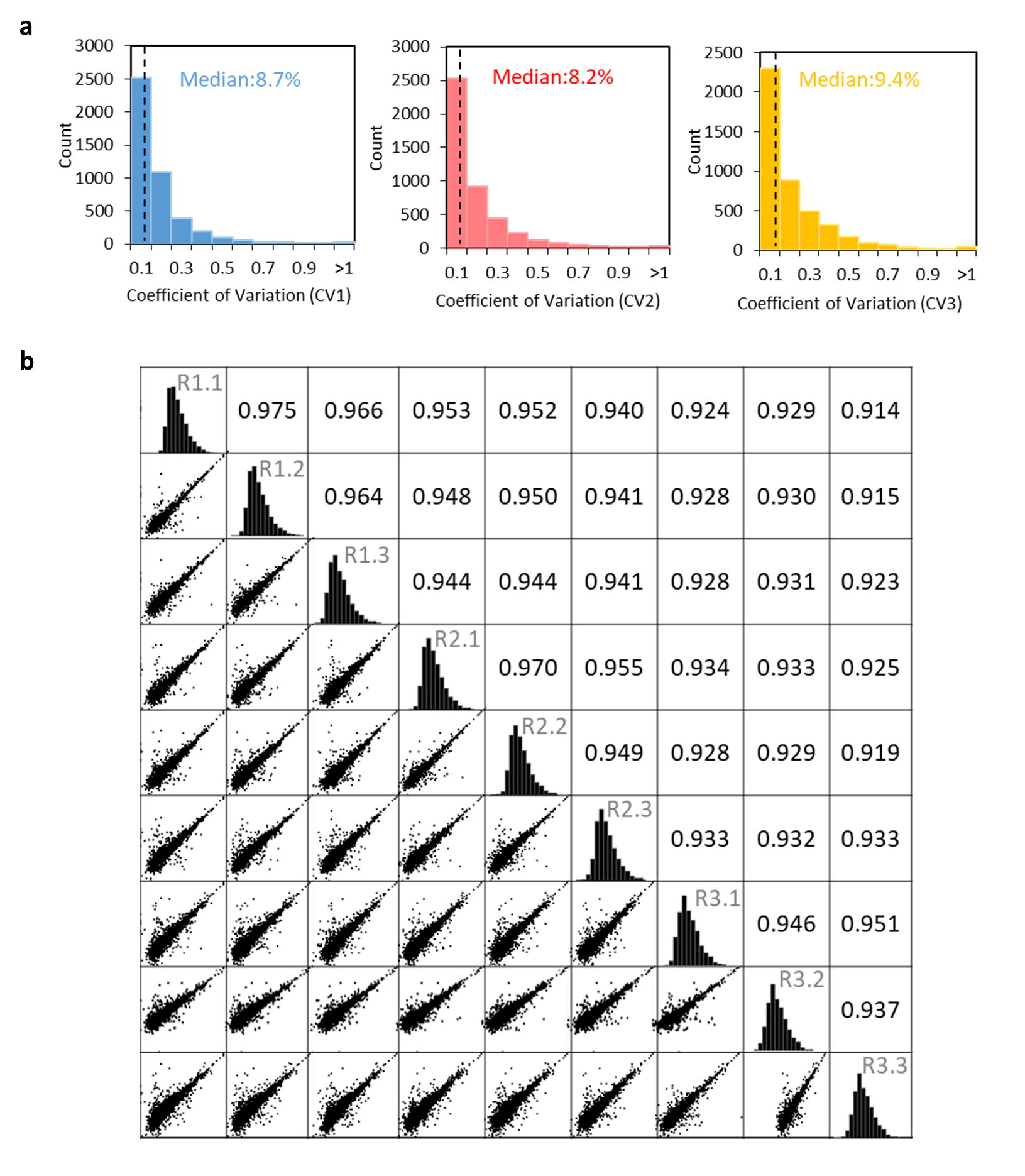


**Figure S1.** **Sample quality control and reproducibility of the DIA data.** (**a)** The median protein coefficient of variations (CV) of the triplicates at three quality control (QC) time points. **(b)** Correlation matrix of the replicates. The replicates are highly correlated (Pearson correlation coefficients>0.90), indicating a high reproducibility in DIA-MS workflow and precision in the quantification of our DIA proteome data set.

**Figure S2.**


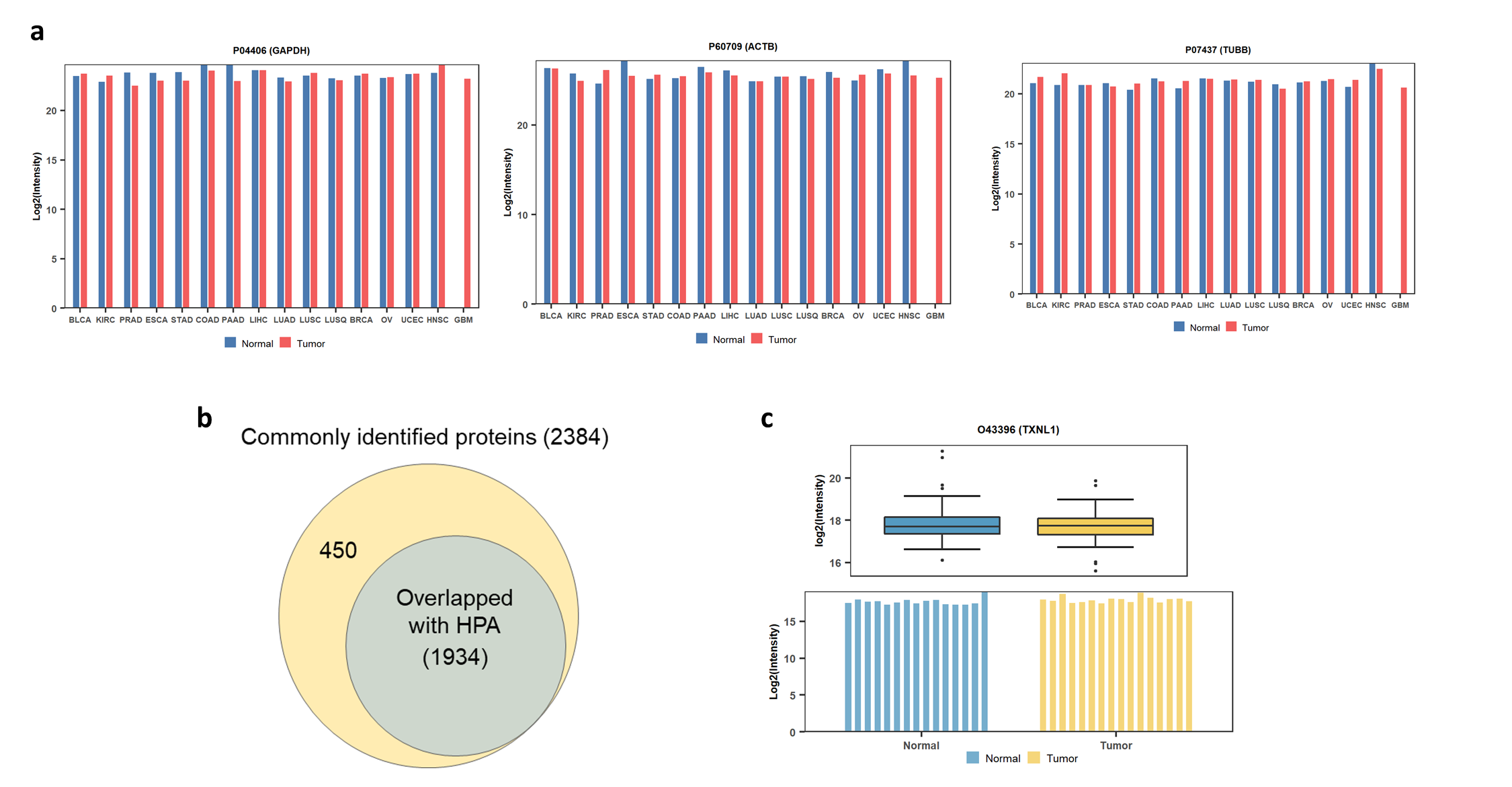


**Figure S2. Characterization of housekeeping proteins.** **(a)** Expression of housekeeping proteins, GAPDH, ACTB, and TUBB, in tumor and normal tissues across different cancer types. **(b)** A comparison of housekeeping proteins identified in this study with the Human Protein Atlas (HPA). **(c)** A relatively consistent expression level of TXNL1 in tumor and normal tissues across different cancer types.

**Figure S3.**


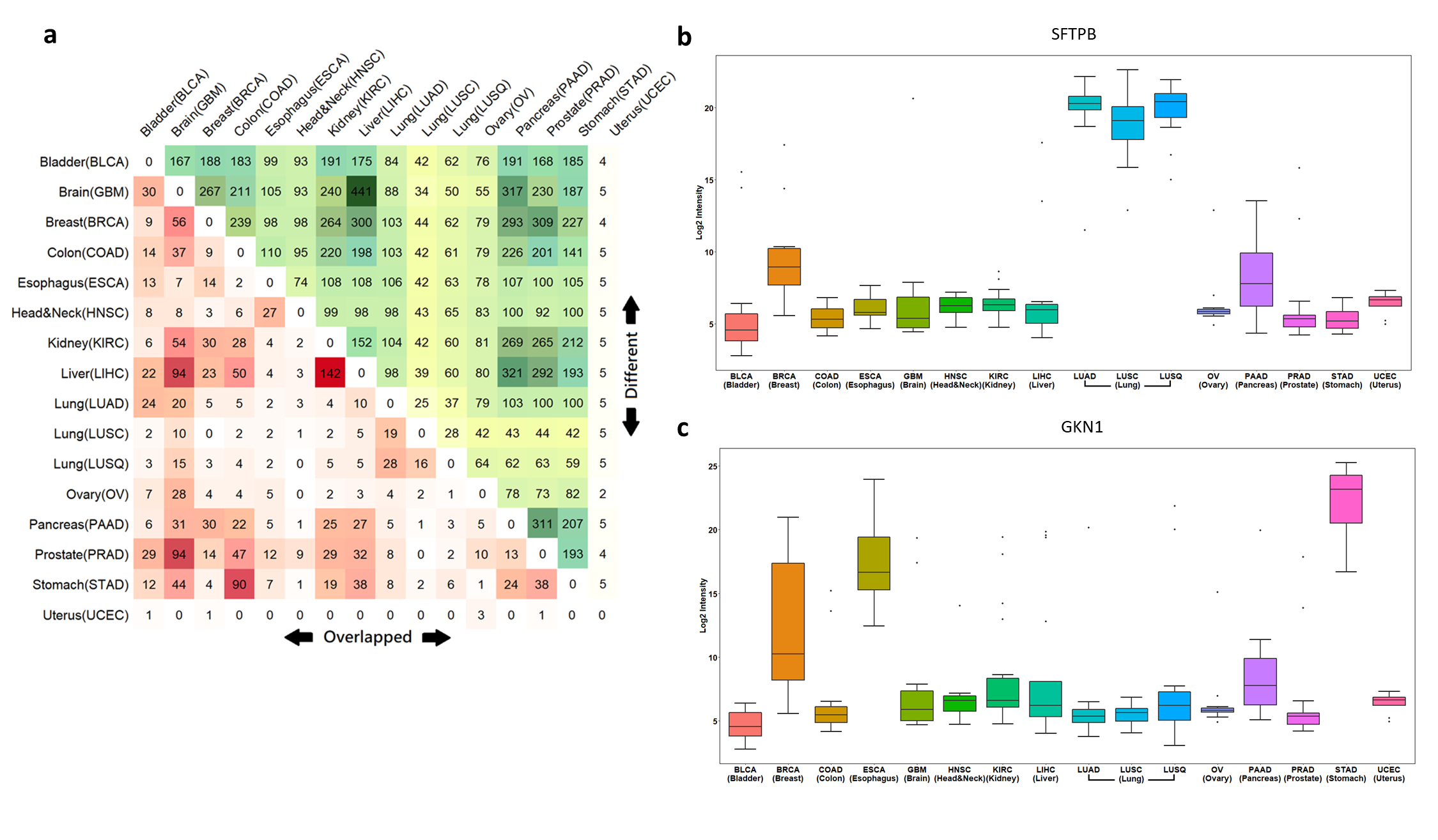


**Figure S3. Proteome analysis of tissue enriched proteins.** **(a)** Pairwise comparison of the tissue enriched proteins in each type of tissue. The bottom triangle shows the total number of commonly identified proteins between two tissues. The upper triangle shows the total number of non-overlapped proteins between two different types of tissue. **(b)** Expression levels of SFTPB across different tissue types. **(c)** Expression levels of GKN1across different tissue types.

**Figure S4.**


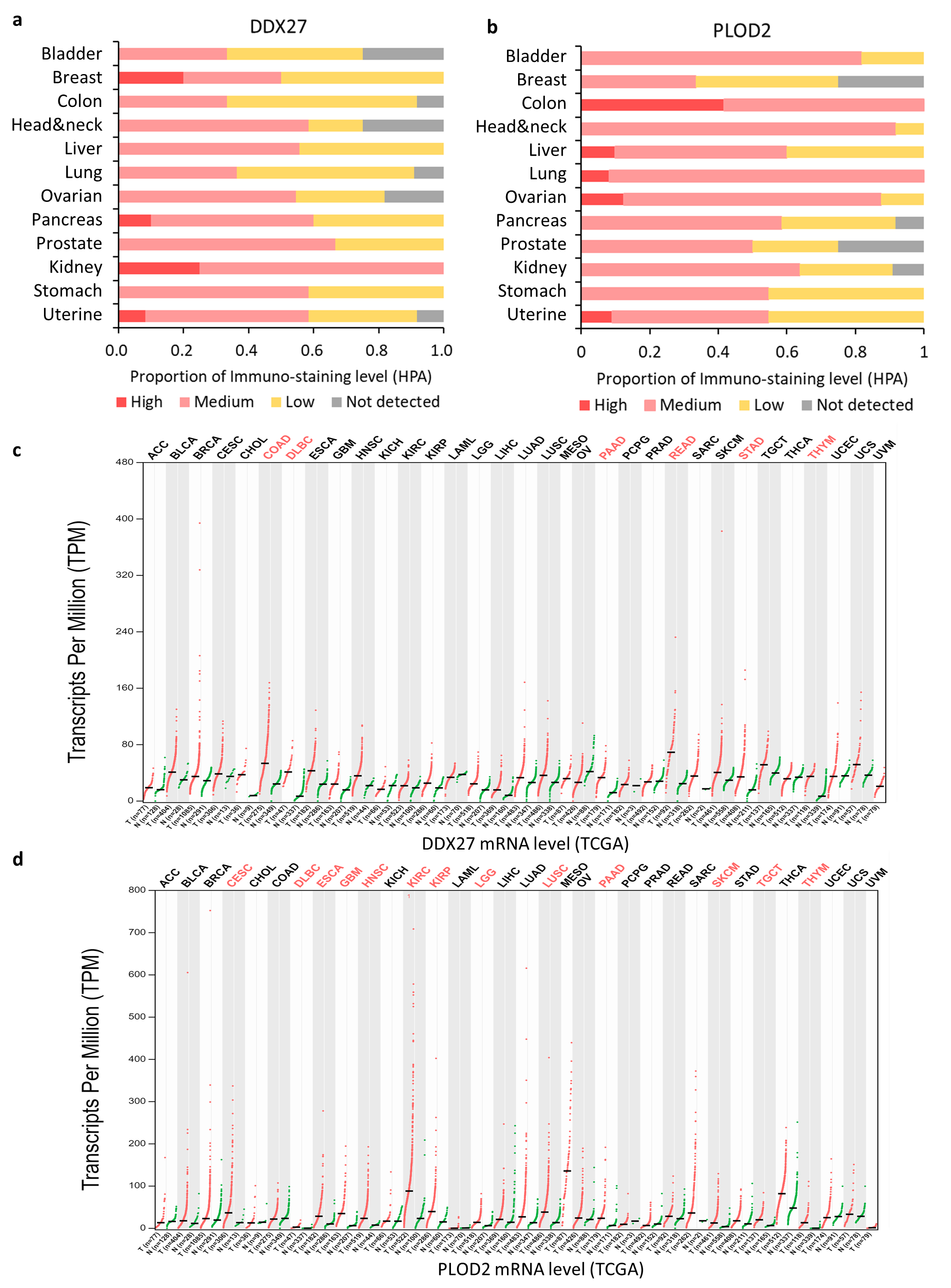


**Figure S4. Protein and mRNA expression from Human Protein Atlas (HPA) and Gene Expression Profiling Interactive Analysis (GEPIA). (a)** Proportions of DDX27 immuno-staining in different cancer types with high staining, medium staining, low staining, or not detected as reported by HPA (version 19.3). **(b)** Proportions of PLOD2 immuno-staining in different cancer types with high staining, medium staining, low staining, or not detected as reported by HPA (version 19.3). **(c)** DDX27 mRNA expression between tumor and normal tissues across multiple cancer types in GEPIA, the green and red color represent the normal and tumor expression, respectively. **(d)** PLOD2 mRNA expression between tumor and normal tissues across multiple cancer types in GEPIA, the green and red color represent the normal and tumor expression, respectively.

**Figure S5.**


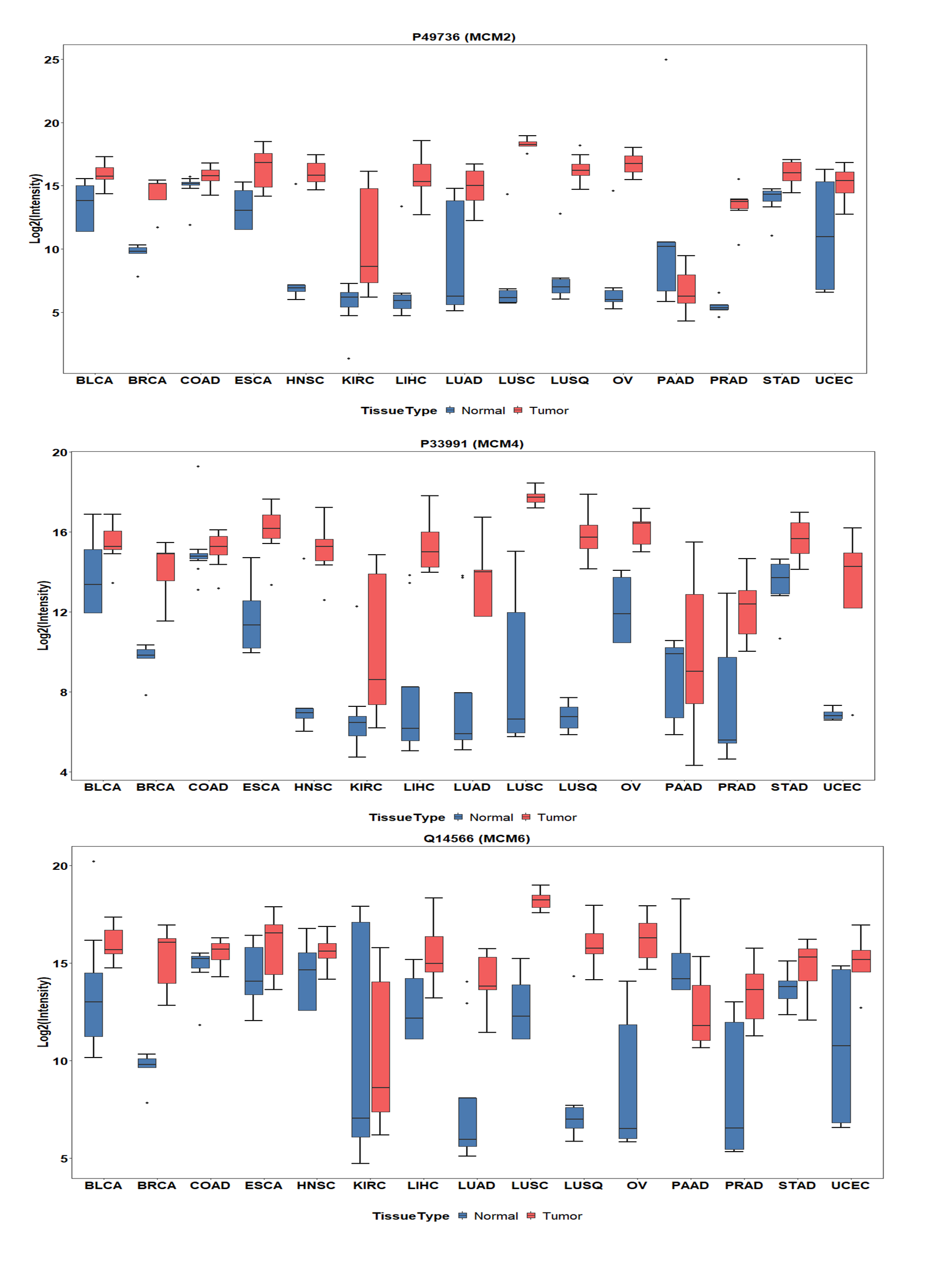


**Figure S5.** Protein expression of MCM2, MCM4 and MCM6 in different cancer types tumor and normal tissues (tumor in red and normal in blue).

**Figure S6.**


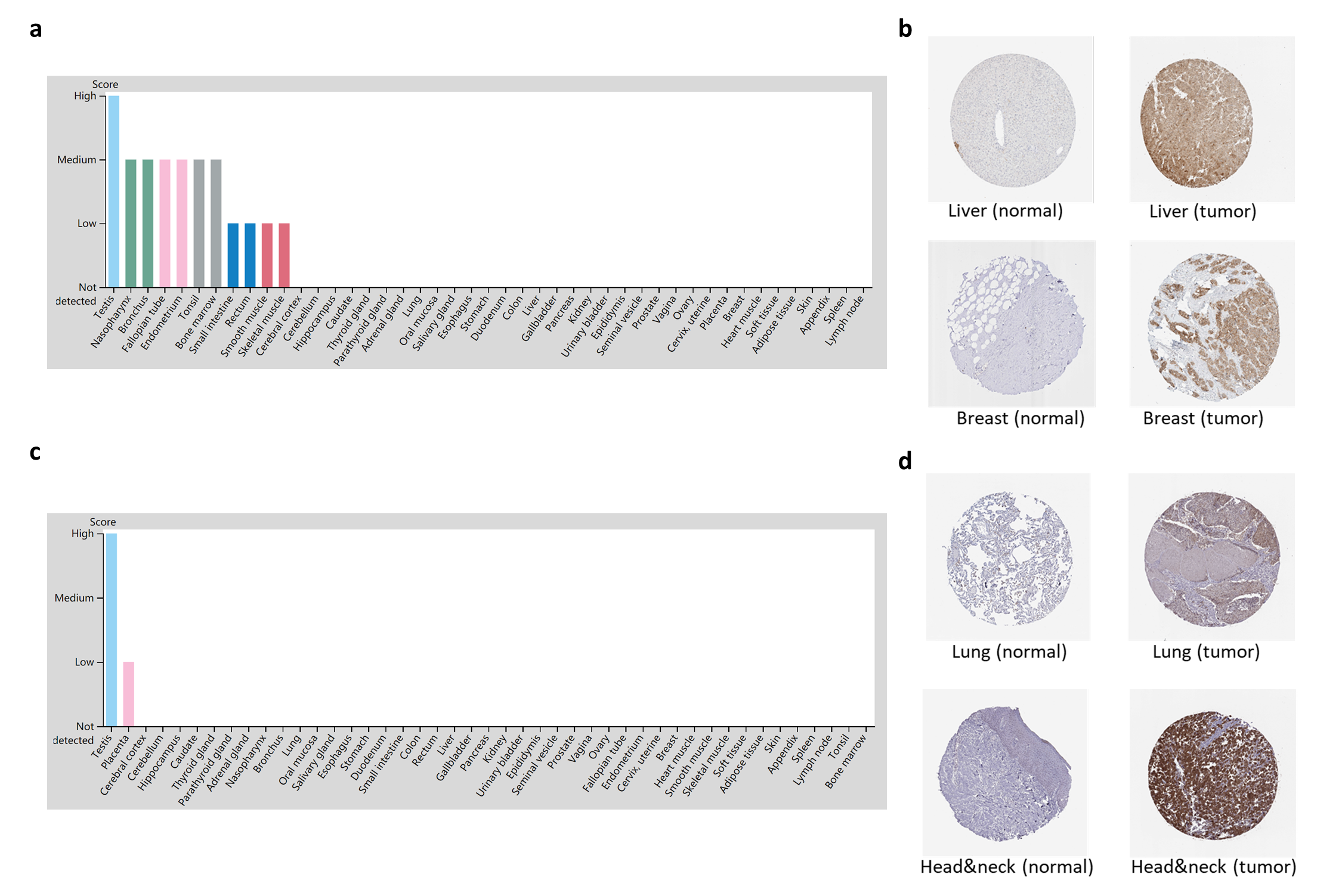


**Figure S6. Protein expression of cancer/testis (CT) antigens according to the Human Protein Atlas (HPA).** **(a)** Expression of sperm associated antigen 1 (SPAG1) is restricted to normal reproductive organs, head and neck, and skeletal muscles. **(b)** Representative immunohistochemical staining images of SPAG1 in normal liver and breast tissues (not detected), and liver and breast cancer (highly expressed). **(c)** Expression of MAGEA4 is restricted to normal testis and placenta. **(d)** Representative immunohistochemical staining images of MAGEA4 in normal lung and head and neck tissues (not detected), and lung and head and neck squamous cell carcinoma (highly expressed).

**Reference**

1. Mertins P, Tang LC, Krug K, Clark DJ, Gritsenko MA, Chen L, et al. Reproducible workflow for multiplexed deep-scale proteome and phosphoproteome analysis of tumor tissues by liquid chromatography–mass spectrometry. Nature protocols. 2018;13(7):1632-61.

2. Rappsilber J, Mann M, Ishihama Y. Protocol for micro-purification, enrichment, pre-fractionation and storage of peptides for proteomics using StageTips. Nature protocols. 2007;2(8):1896.
